# Impact on the Quality of Life When Living Close to a Municipal Wastewater Treatment Plant

**DOI:** 10.1155/2016/8467023

**Published:** 2016-06-07

**Authors:** A. Vantarakis, S. Paparrodopoulos, P. Kokkinos, G. Vantarakis, K. Fragou, I. Detorakis

**Affiliations:** Environmental Microbiology Unit, Department of Public Health, Medical School, University of Patras, University Campus, 26500 Patras, Greece

## Abstract

The objective of the study was to investigate the impact on the quality of life of people living close to a municipal wastewater treatment plant. A case control study, including 235 inhabitants living within a 500 m radius by a municipal wastewater treatment plant (cases) and 97 inhabitants living in a different area (controls), was conducted. A standardized questionnaire was self-completed by the participants which examined the general health perception and the overall life satisfaction. Also, the concentration of airborne pathogenic microorganisms in aerosol samples collected around the wastewater treatment plant was investigated. Significant risk for symptoms such as headache, unusual tiredness, and concentration difficulties was recorded and an increased possibility for respiratory and skin diseases was reported. A high rate of the cases being irritable and moody was noticed. Significantly higher gastrointestinal symptoms were also reported among the cases in relation to the controls. The prevalence of pathogenic airborne microorganisms originating from the wastewater treatment plant was reported in high numbers in sampling points close to the wastewater treatment plant. More analytical epidemiological investigations are needed to determine the cause as well as the burden of the diseases to inhabitants living surrounding the wastewater treatment plant.

## 1. Introduction

Air quality and its pollution (physical, chemical, and biological) significantly influences the health and good living of humans, animals, or plants inhabiting it [1, 2]. Despite the fact that the air is an unfavourable environment for microorganisms to grow, it is merely a place which temporarily occupy and move in. The air is very often called “transport environment” because microorganisms may be present and often can be transported over considerable distances [1]. Microorganisms move in the air as a consequence of wind movement, which “sweeps” them away from various habitats and surroundings (soil, water, waste, plant surfaces, animals, and other), or are introduced during the processes of sneezing, coughing, or sewage aeration [2].

Wastewater treatment plant (WTP), due to its working conditions, is considered as a major source of aerosols and may constitute an important health risk for plant workers as well as the surrounding inhabitants [2–5]. Various bacterial and fungal communities have been isolated from all types of aerobic and anaerobic WTPs [6]. Several studies have shown that bacteria contained in droplets of WTPs were 10–1000 times more than that in a water source, depending on the droplet size [3]. A number of atmospheric factors such as temperature, wind velocity, smog, and specific humidity influence the aerosol spread as well as the ability of microorganisms to survive in the air. At very low humidity and high temperature, microbes face dehydration, whereas high humidity may give cells protection against the solar radiation [3, 4, 7]. It has also been reported that UV radiation, oxygen content, specific ions, various pollutants, and air-associated factors are also responsible for the decrease of the biological activity in a WTP [7, 8].

Bioaerosols may contain different types of microorganisms such as viruses, pathogenic bacteria, and fungi, capable of causing skin, digestive system, respiratory, and nervous system diseases and human allergies [9]. Specifically, bioaerosols emitted by WTPs can impact the air quality. In the past, microbial concentrations in the surrounding air from the aeration tanks of WTPs, at different heights and different distances, have been reported [10–12].

Waste management facilities generate atmospheric emissions and liquid effluent, which may be hazardous to human health. The potential health hazards related to WTP aerosols are documented commonly for occupational exposure. Effects including respiratory and digestive symptoms have been reported in workers exposed to particulate matter and bioaerosols [9]. Similar health problems may occur in people living near such plants who may be exposed to this release. To guide the implementation of waste management policies, decision-makers need information about their potential effects on public health.

In the city of Patras, south western Greece, a municipal wastewater treatment plant receiving domestic sewage from approximately 250,000 citizens is located in a densely inhabited area. The WTP effluents flow to the Patraikos gulf through a submarine pipe delivering the treated effluents in approximately 100 m from the coastline. Within a radius of 100–500 m around the WTP, 800 to 1000 inhabitants are permanently living. In order to assess the impact on the quality of life of citizens living close to the WTP, an observational case control study, as well as a microbiological analysis of air close to the living areas, was performed. It is the first time that such an observational survey has been performed in Greece. It is one of the very few studies combining microbiological and epidemiological data in an area close to a wastewater treatment plant.

## 2. Materials and Methods

The Patras' wastewater treatment plant (WTP) has a mean inflow of 45,000 m^3^/d receiving municipal waste from 250,000 inhabitants. It is a secondary WTP which includes indoor pretreatment with screens and coarse bubble aerated grit clambers, outdoor primary and secondary settling tanks, outdoor chlorination, and indoor sludge processors with belt filter presses.

### 2.1. Study Population

**Figure 1:**
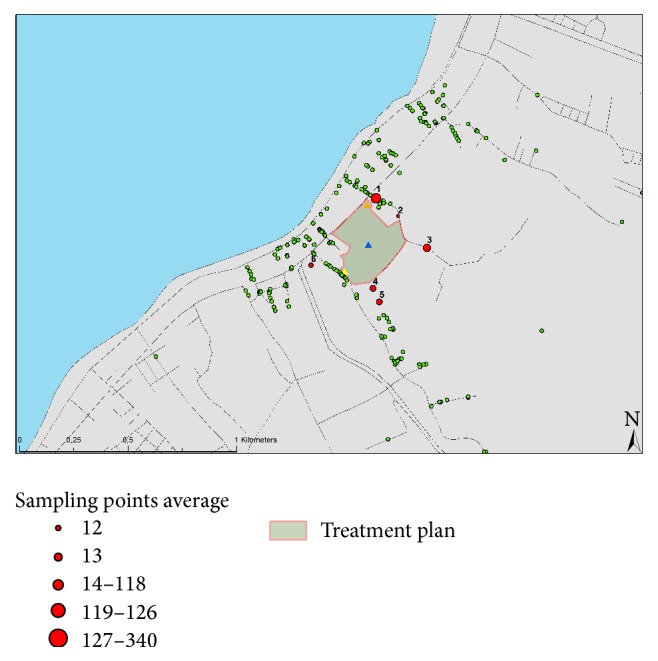
Microbiological sampling stations and results as well as questionnaire locations in a perimeter of a radius of 500 m.

The study population was comprised of inhabitants living in the surrounding area of the WTP (up to 500 m radius) considered as cases. A case included any resident, living permanently for more than eight hours per day in an area (<500 m) from the WTP. As a control was considered a resident living permanently in an area located more than 5 km from the WTP. The participants, cases and controls, were matched according their demographic, socioeconomic, ethnic, and occupational background. Inclusion criteria in the study were the permanent residency in the region, the age above 18 years, and the agreement to complete the questionnaire. Cases travelled and stayed abroad as well as individuals who were working far from their house for more than 10 hours every day or who resided in the regions for less than a year were excluded from the study (Figure 1).

### 2.2. Study Design

Study participants completed a structured self-administered validated questionnaire distributed at their homes [13]. Participation was on a voluntary basis. The questionnaire was divided into three parts and contained 60 questions.

The first part (23 questions) assessed baseline characteristics including sociodemographic variables such as age, sex, family status, education, occupation, place of work, socioeconomic status, life habits (tobacco and/or alcohol), and general health perception. The health status was indicated by a distinction between poor and good health. The exact wording and response option of current health question is consistent with recommendations of the WHO [14] and the EURO-REVES 2 group [15]. Participants were asked, “In general how would you describe your current health status.” Those who responded “very good” “good” or “satisfying” were considered to be in good health, while those who responded “poor” or “bad” health were considered to be in poor health.

The second part (10 questions) was concerned with the medical history of participants: presence and frequency of gastrointestinal and respiratory symptoms, joint pains, and central nervous system symptoms (including headache, unusual tiredness, and concentration difficulties). Special questions were related to physician diagnosed allergy, eczema, and asthma. The grouping of symptoms was as follows: respiratory (asthma, chronic bronchitis, and chronic sinusitis), gastrointestinal (abdominal pain and bloating, nausea, vomiting, diarrhoea, constipation, and jaundice), skin (skin rash, ulcer on the skin) or systemic (headache, fever, chest pain or discomfort, muscle spasms, chills, irritability, insomnia, fatigue, weakness, and vague general discomfort or feeling of illness), allergies at last year (drugs, powder, materials, etc.), blood diseases (thalassemia, leukaemia), and musculoskeletal diseases (osteoporosis, backache).

The third part (27 questions) related to health-related quality of life and overall life satisfaction. The questions assessed the occurrence of four subjective physical and psychological health complaints, namely, being moody, irritable, bad tempered, and unhealthy.

The questionnaire has been piloted into 20 respondents before its use. Also, a test-retest system was used to assess the reproducibility of the responses, 20 subjects being required to complete a second questionnaire after one-month interval.

### 2.3. Air Sampling Strategy

Sampling of aerosols was performed once a week for four consecutive weeks during summer period, from 6 sampling stations in an area of 500 m radius around of the Patras' WTP. The sampling points were recorded using a GPS instrument (Magellan Explorist, Aachen, Germany). Three samplings were performed at different times of each sampling day (morning 8.30 a.m., afternoon 18:00 p.m., and night 22:00 p.m.) from each sampling station, in order to monitor the presence of microorganisms during the whole day. Microbiological investigation was carried out during ordinary workdays when biological treatment plant was normally working. Throughout the studied period, during air sampling, air temperature, relative humidity, wind direction and speed, and solar radiation were measured.

During each sampling period, an average of three readings of humidity and temperature was recorded. The temperature (expressed in °C) and the relative humidity (expressed in %) were measured with a portable instrument (Opus 10 Lufft, Germany).

Aerosol samples were collected using a sampler (International PBI Surface Air System, SAS, Italy). Petri dishes (55 mm diameter) containing 25 mL of Tryptic Soy Agar medium, (TSA Merck, Darmstadt, Germany) were placed into a special support of the sampler. The sampling flow rate was 90 L/min. A 15 min sampling time (volume of air > 1000 L) was used and samples were transported to the laboratory within 2 hours for further analysis. The air sampler was disinfected with 70% denaturized ethanol (CarloErba, Milano, Italy) after each sampling. Petri dishes were incubated at 36°C (±1°C) for 24 hours. After the incubation period, one experienced analyst enumerated bacterial colonies on each plate based on their cell morphology. Bacterial colonies were differentiated on the basis of colony morphology, Gram staining, and catalase and oxidase test. Following Gram staining, at least three characteristic and distinctive Gram negative colonies from each plate were identified using the API system (bioMerieux, Marcy I'Etoile, France). Also* Staphylococcus* spp. (ISO 6888-2:1999),* Enterococcus* spp. (ISO 7899-02:2000), and total coliforms/*Escherichia coli* (ISO 9308-1:2000) were identified. The concentration of airborne bacteria was finally expressed as colony forming units (CFU)/m^3^. No major environmental problems were reported at the sampling stations during the survey period. Concentrations on a limited number of days were considered representative of the annual microbial concentrations.

### 2.4. Statistical Analysis

All statistical analysis was conducted with SPSS 21.0, while, for the mapping, Arc-GIS 9.2 software was applied (ESRI, USA). Data were analysed using descriptive statistics (Chi-test) and logistic regression to determine odds ratios and statistical significance. Differences in selected demographic variables, as well as smoking and health status, between the cases and the controls were evaluated by the Chi-square test. Student's *t*-test was used to evaluate continuous variables, including age and pack-years of cigarette smoking. Unconditional multivariate logistic regression analysis was employed to examine the association of living near the WTP and the development of health problems by estimating odds ratio (ORs) and 95% confidence intervals (95% CI).

The baseline characteristics were compared between the two study groups using the Chi-square and *t*-tests. Multivariate analyses, using a logistic regression model, were conducted to compare the prevalence of the investigated chronic diseases, adjusted for demographics and health-related habits. Comparisons of the questionnaire components were performed with Mann-Whitney *U* test, and for multivariate analysis linear regression models were computed. The independent variables for the models were demographics, health-related habits, and chronic conditions.

Nonparametric statistics were usually used to test for relationships between pathogen concentration and other factors, because total airborne bacteria (TAB) were not normally or log-normally distributed. A nonparametric Mann-Whitney test was used to determine whether there were significant differences in microorganism concentrations based on the factors evaluated in this study. Spearman's correlation analyses were used to examine the relationship between microorganism concentration and the other factors. A nonparametric Kruskal-Wallis test and analysis of variance were also performed to determine whether there were differences in microorganism concentration by sampling location and date. A *P* value lower than 0.05 was considered significant, for all statistical analyses. All values are expressed as mean (SD).

## 3. Results

### 3.1. Questionnaire Validation

#### 3.1.1. Acceptability

Ten subjects (4.2%) refused to complete the questionnaire.

#### 3.1.2. Feasibility

Three subjects (1.3%) failed to complete the questionnaire owing to poor eyesight.

The average time for completion was 15 minutes (range 10 to 20 minutes). The completion rate for the questionnaire was 90% of all questions.

#### 3.1.3. Reproducibility

In both groups (case control) the test-retest study showed that only one answer (1.75%) was altered in one questionnaire (0.4%).

### 3.2. Epidemiological Survey Study

A structured questionnaire was administered to the 235 cases and 97 controls (Table 1) to obtain information on demographics, knowledge of their general health status, and determination of frequency of physical symptoms that they have experienced in the study period. All respondents were asked to give complete answers. The participants (cases and controls) self-filled in the study questionnaire and returned it anonymously indicating only the address (Figure 1).

The 86.8% of the cases were staying at home for more than 8 hours. The smoking habits of cases and controls were reported in Table 2. The 26.8% of the cases considered their healthy status as nonsatisfactory (average and bad) compared to 17.8% of the controls (*P* < 0.001). A statistically significant negative relationship (*r* = −0.58, *P* < 0.001) between cases living near the WTP and their general perception about their health status was also noted.

The incidence of allergies among the cases reached the 27.8% and most of them were allergic to dust and pollen. Questionnaires showed that 8.7% had iron deficiency anaemia and 27.5% were suffering from migraine headache. 7.2% had asthma and 12.9% gastritis. Dermatitis occurred in 9.3% and the medicine use reached 41.1%. The mood as well as the perception about their health between cases and control is shown in Table 3.

There was no increased rate of gastrointestinal disorders or myoskeletal diseases. Similarly, there were no significant increases in the rates for respiratory, allergic, and blood diseases. However, there was a significant increase in the rate of neural disorders (Table 4). The frequency of the symptoms is reported in Table 5. Almost all cases (79.6%) complained about strong odors coming from the WTP during the evening (40.4%), during the afternoon (20.8%), during the midday (10.7%), and during the morning (28.1%). Odors were more intense in spring (28%) and summer (36.4%) (Table 6). Cases emphasized problems due to the presence of the WTP as follows: odors (50.9%), air suspensions (1.1%), and different health problems (6.3%). It should be mentioned that 72.8% of the residents found the presence of the WTP indispensable, but 17.4% believed that it was dangerous for their health.

### 3.3. Air Microbiological Study

**Figure 2:**
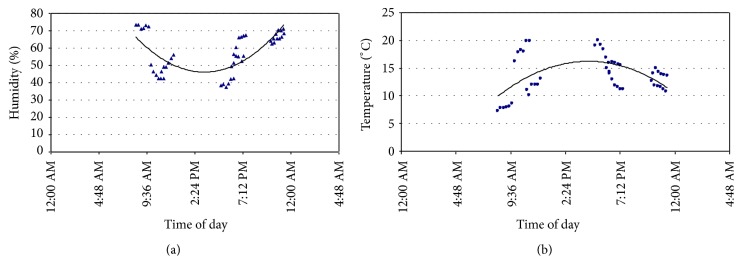
Measurements of humidity (a) and air temperature (b) during the study period.

Forty-seven (47) measurements of temperature (°C) and humidity (%) were carried out during the sampling period (Figure 2). The mean temperature was 13.6°C varying from 7 to 20°C and the mean relative humidity was 57.3%, varying from 38% to 74%. During the evening sampling campaigns, the ambient temperature ranged from 10.8 to 14.9°C and the relative humidity was approximately 67%.

**Figure 3:**
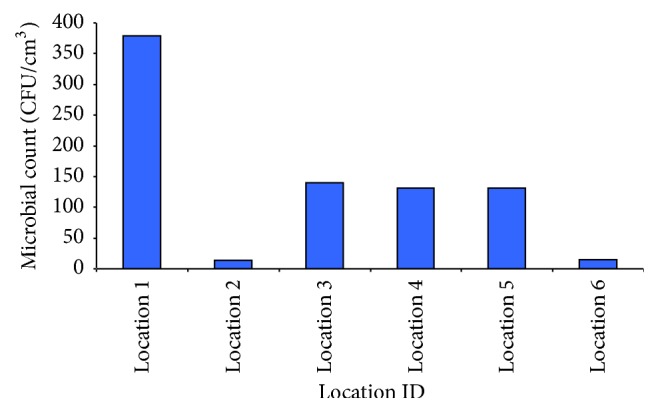
Average microbial count per sampling location (CFU/m^3^).

Eighty-three (83) randomly selected isolated bacterial colonies were isolated and identified. Depending on their Gram staining, the microorganisms were initially mainly characterized as cocci (79.5%), as Gram positive bacilli (7.2%), and as Gram negative bacilli (13.3%). Summarized microbiological data are shown in Table 7. Twenty-four strains (29%) were identified as* Staphylococcus aureus*, 30 (36%) as* Streptococcus* spp., 4 (4.9%) as* Enterococcus* spp., and 7 (8.5%) as* Escherichia coli*. Eighteen (21.7%) strains of bacteria could not be typed. The detected loads of airborne microorganisms at the six different sampling stations were, in general, low, but a few higher concentrations were found at the two closest sampling stations, (Locations number 1, number 3). Concentrations of airborne bacteria at each sampling station are shown in Figure 3. Among the sampling locations, Location 1 had the highest concentration of culturable airborne bacteria, with 340.89 CFU/m^3^. As the distance increased from the center of the WTP, the concentration of culturable bacteria gradually decreased. Mean concentrations were found lower, while the distance from the center of the WTP was increased more than 800 m. None of the collected air samples was found positive for* Salmonella* spp. 

**Figure 4:**
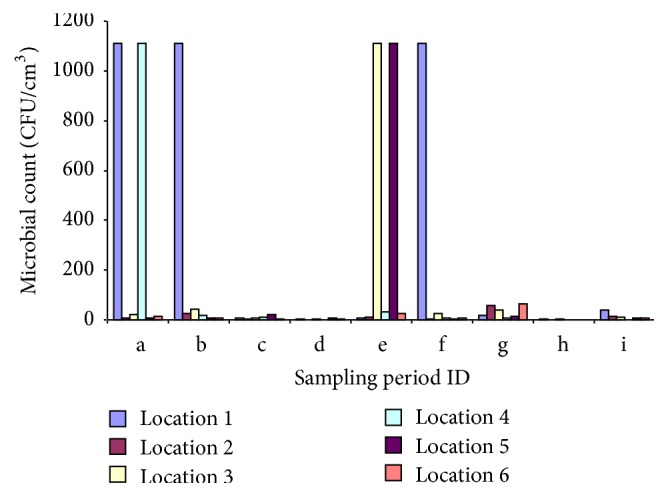
Average microbial count per sampling station (location) and sampling period (CFU/m^3^).

**Figure 5:**
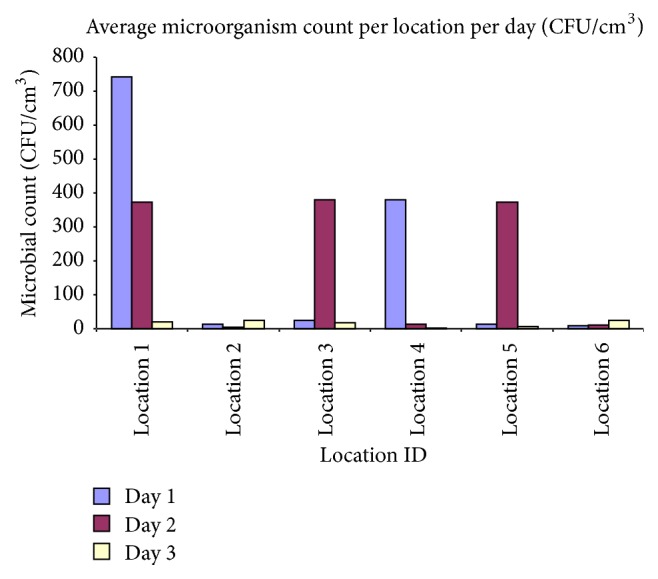
Average microbial count per sampling station (location) and sampling period (CFU/m^3^).

Triplicate samples of bacteria (*Streptococcus* spp.,* Enterococcus* spp.) were collected at each sampling time. The airborne microbial concentrations (CFU/m^3^) corresponding to the three campaigns in all locations are summarized in Figure 4. The average microbial load per sampling location per day (CFU/m^3^) is shown in Figure 5, respectively.

## 4. Discussion

In the present study, the impact on the quality of life of inhabitants living close to a WTP as well as the evaluation of the air microbiological quality was reported.

Air microbiological analyses have commonly been conducted close to sewage treatment plants [3]. Sawyer et al. [12] measured concentrations of 126–4840 bacterial CFU/m^3^ at different heights above the water surface of the aeration tank of wastewater treatment plants. Brenner et al. [10] recorded concentrations of 86–7143 bacterial CFU/m^3^ air at a distance of 25 m from the surface of an aeration basin well. Another study showed that the air densities of total aerobic bacteria-containing particles, total coliforms, faecal coliforms, faecal streptococci, total count bacteria, and coliphages increased significantly within the perimeter of the plant during operation of the wastewater treatment plants [11]. Other studies have shown that a percentage of the emitted bacterial contamination can be transported over considerable distances [10]. In our study the highest microbial numbers have been reported in the locations close to the WTP.

In order to evaluate the results of the air microbiological analyses, it should be considered that the recorded microbial loads represent only a “picture” of the sampling time. In connection, with the physicochemical properties of the air, the degree of contamination at a given point can significantly change within a few minutes [16]. An important issue of the study was the season in which the study was performed, which is known to play a significant role in the dispersion of aerosols and odors in the air, as well as microbes, especially during specific seasons of the year. Complaints related to the odors were increased during the summer months and especially during early the morning or evening, when the percentage of humidity was higher at the sampling stations. It is suggested that the seasonal variations of bacterial loads might be related to the contingent meteorological conditions (humidity, temperature) and to the intrinsic sensitiveness of different bacteria genera to these factors [17].

Some WTPs produce higher concentrations of bioaerosols compared to others. In previous studies, using personal samplers, it was shown that sewage treatment plant employees that have a higher incidence of headache, tiredness, and nausea were exposed to culturable bacteria. Exposure to rod-shaped bacteria and total number of bacteria was significantly higher in workers reporting headache during work than in workers not reporting headache [11].

A few studies have shown that blood tests of workers who were subjected to aerosol inhalation indicated an increased level of antibodies against Gram negative bacteria and intestinal viruses. The condition has been described as “the sewage worker's syndrome,” which has a viral origin and manifests itself with a despondency, overall weakness, catarrh, and fever [11, 18]. Main characteristics of the disease included general malaise, weakness, acute rhinitis, and fever [19], accompanied by gastrointestinal symptoms. In accordance with these studies, we recorded increased odds for the inhabitants who lived near the WTP to develop neurological and myoskeletal symptoms at 3.37 and 1.98 times, respectively. Moreover, sewage workers and those who live in the vicinity of a WTP have higher morbidity with intestinal and respiratory system illnesses [11, 20]. In order to ensure public health, health of workers, and good quality of life, it is necessary to determine the composition and concentration of microorganisms in the air. Skin contact, ingestion, and inhalation are the three major routes of exposure to airborne particles [20]. Microorganisms that are associated with intestinal infections such as* Salmonella* spp. and enteric viruses are thought to be transmitted through inhalation [4, 21].

Also, a nationwide survey in Sweden showed that an increased risk for headache, concentration difficulties, unusual tiredness, and head heaviness was reported in workers compared to the controls [18]. Similarly, in our study, feelings like tiredness and sickness were more reported by the cases compared to the controls. Interestingly, our study showed an increased rate in mental disorders to the population living near the WTP. There was no significant correlation of the WTP and the occurrence of gastrointestinal or myoskeletal symptoms to the residents. Also, this study showed no significant correlation concerning gastrointestinal, allergic, and respiratory symptoms although the study sample of the controls was rather small due to the refusal of controls (people in the city) to participate in the study.

In our study, there is a significant presence of possible pathogenic microorganisms in the aerosols close to WTP and this concentration depended on the distance. There is indication of the burden of microorganisms in air according to the distance of the inhabitants. To establish aerosols impact on the human health, more extensive studies are needed including medical examinations in inhabitants. Such studies have not been performed to the area of the WTP.

In order to lower the impact for public health, in areas like this, retaliatory preventive measures should be taken by the authorities in order to protect inhabitant's health. Such measures could be considered the tree growing around the WTP as well as the appropriate function of the WTP with protective equipment for the aerosols.

## Figures and Tables

**Table 1:** Demographic characteristics of the study population.

Sample characteristics	Cases (235)		Controls (97)		*P* value
	*N*	%	*N*	%	
Sex					0.074
Male	107	45.5	33	34	
Female	126	53.6	61	62.9	

**Table 2:** Comparison between cases and controls concerning smoking habits.

Sample characteristics	Cases (235)		Controls (97)		OR	CI	*P* value
	*N*	%	*N*	%			
Smoker	111	47.2	31	32	1.849	1.120–3.052	**0.015**
Previous smoker	31	13.2	24	24.7	0.707	0.372–1.345	0.290
Years of smoking							**0.001**
<5 years	5	2.1	9	9.3			
5–10 years	29	12.3	13	13.4			
>10 years	97	41.3	23	23.7			
Quantity of cigarettes							0.502
<10 cig.	26	11.1	11	11.3			
10–20 cig.	74	31.5	19	19.3			
>20 cig.	32	13.6	11	11.3			

**Table 3:** Frequency of feelings from the inhabitants close to the WTP, compared to the controls.

Sample characteristics	Cases (235)		Controls (97)		*P* value
	*N*	%	*N*	%	
Mood	126	53.6	60	63.8	0.058
Freq. of having bad mood (>2/week)	54	42.9	17	28.4	**<0.05**
Angry	135	57.4	52	58.4	0.873
Freq. of being angry (>2/week)	64	47.4	19	36.6	**0.05**
Tired	154	65.5	67	70.5	0.382
Freq. of being tired (>2/week)	91	59.1	37	57.2	0.904
Sick	36	15.3	19	21.1	0.213
Freq. of being sick (>2/week)	24	68.6	4	22.3	**0.001**

**Table 4:** Health symptoms associated with the distance living of WTP.

Symptoms/diseases	Cases (235)		Controls (97)		OR	CI	*P* value
	*N*	%	*N*	%			
Blood	29	12.3	10	10.3	1.37	0.56–3.38	0.601
Neural	102	43.4	18	18.6	4.06	1.82–9.04	**0.001**
Respiratory	39	16.6	21	21.6	0.82	0.34–1.96	0.276
Gastrointestinal	55	23.4	28	28.9	1.07	0.52–2.23	0.296
Skin	29	12.3	13	13.4	0.910	0.45–1.83	0.791
Myoskeletal	66	28.1	16	16.5	1.52	0.69–3.41	**0.026**
Allergies	65	27.8	37	43	0.77	0.38–1.57	**0.009**

**Table 5:** Frequency of symptoms and medical consultation.

Sample characteristics	Cases (235)		Controls (97)		*P* value
	*N*	%	*N*	%	
Freq. of gastrointestinal symptoms (>1/6 months)	38	16.2	31	36.1	**0.001**
Medical consultation	21	13.3	13	24.1	0.062
Freq. of respiratory symptoms (>1/6 months)	45	19.2	23	28.4	0.145
Medical consultation	40	25	22	42.3	**0.017**
Freq. of allergy symptoms (>1/6 months)	59	25.6	17	21.3	0.751
Medical consultation	50	31.4	10	25.6	0.480

**Table 6:** Odors existence and frequency of occurrence (235 cases).

Odors existence	**187**	**79.6**%
Frequency of odors (>3 times/month)	145	61.7%
Odors daily timetable		
Early hours	92	28.1%
Midday hours	35	10.7%
Afternoon hours	68	20.8%
Evening hours	132	40.4%
Odors yearly timetable		
Spring	135	28%
Summer	176	36.4%
Autumn	86	17.8%
Winter	86	17.8%

**Table 7:** Types of identified bacteria.

Microorganisms	Isolated bacteria
*Staphylococcus aureus*	24 (28.92%)
*Streptococcus* spp.	30 (36.14%)
*Enterococcus* spp.	4 (4.82%)
*Escherichiacoli*	7 (8.43%)
